# Dendritic cells, engineered to overexpress 25-hydroxyvitamin D 1α-hydroxylase and pulsed with a myelin antigen, provide myelin-specific suppression of ongoing experimental allergic encephalomyelitis

**DOI:** 10.1096/fj.201601243R

**Published:** 2017-03-31

**Authors:** Chih-Huang Li, Jintao Zhang, David J. Baylink, Xiaohua Wang, Naga Bharani Goparaju, Yi Xu, Samiksha Wasnik, Yanmei Cheng, Edmundo Carreon Berumen, Xuezhong Qin, Kin-Hing William Lau, Xiaolei Tang

**Affiliations:** * Division of Regenerative Medicine, Department of Medicine, Loma Linda University, Loma Linda, California, USA;; † Department of Emergency Medicine, Chang-Gung Memorial Hospital, Linkou Medical Center, Taoyuan, Taiwan;; ‡ Graduate Institute of Clinical Medical Sciences, School of Medicine, Chang-Gung University, Taoyuan, Taiwan;; § Institute of Medical and Pharmaceutical Sciences, Zhengzhou University, Henan, China;; ¶ Division of Infectious Disease, Jinan Infectious Disease Hospital, Shandong University, Jinan, China;; ‖ Yue Yang Hospital of Traditional Chinese and Western Medicine, Shanghai University of Traditional Chinese Medicine, Shanghai, China;; # Musculoskeletal Disease Center, Jerry L. Pettis Memorial Veterans Affairs Medical Center, Loma Linda, California, USA

**Keywords:** multiple sclerosis, regulatory T cells, T_reg_, 1,25(OH)_2_D, foxp3

## Abstract

Multiple sclerosis (MS) is caused by immune-mediated damage of myelin sheath. Current therapies aim to block such immune responses. However, this blocking is not sufficiently specific and hence compromises immunity, leading to severe side effects. In addition, blocking medications usually provide transient effects and require frequent administration, which further increases the chance to compromise immunity. In this regard, myelin-specific therapy may provide the desired specificity and a long-lasting therapeutic effect by inducing myelin-specific regulatory T (T_reg_) cells. Tolerogenic dendritic cells (TolDCs) are one such therapy. However, *ex vivo* generated TolDCs may be converted into immunogenic DCs in a proinflammatory environment. In this study, we identified a potential novel myelin-specific therapy that works with immunogenic DCs, hence without the *in vivo* conversion concern. We showed that immunization with DCs, engineered to overexpress 25-hydroxyvitamin D 1α-hydroxylase for *de novo* synthesis of a focally high 1,25-dihydroxyvitamin D concentration in the peripheral lymphoid tissues, induced T_reg_ cells. In addition, such engineered DCs, when pulsed with a myelin antigen, led to myelin-specific suppression of ongoing experimental allergic encephalomyelitis (an MS animal model), and the disease suppression depended on forkhead-box-protein-P3(foxp3)^+^ T_reg_ cells. Our data support a novel concept that immunogenic DCs can be engineered for myelin-specific therapy for MS.—Li, C.-H., Zhang, J., Baylink, D. J., Wang, X., Goparaju, N. B., Xu, Y., Wasnik, S., Cheng, Y., Berumen, E. C., Qin, X., Lau, K.-H. W., Tang, X. Dendritic cells, engineered to overexpress 25-hydroxyvitamin D 1α-hydroxylase and pulsed with a myelin antigen, provide myelin-specific suppression of ongoing experimental allergic encephalomyelitis.

Multiple sclerosis (MS) is a chronic inflammatory disorder in the CNS. The disease afflicts more than 2.5 million individuals worldwide (1). In addition, data suggest that the global prevalence and incidence rate of MS are increasing. It is believed that the disease is due to immune-mediated damage of myelin sheath, leading to demyelination and secondary loss of axons and neurons (2). In this regard, an array of FDA-approved medications is available for reducing the frequency and severity of clinical attacks, as well as the number of new lesions in the CNS, by blocking immune responses in the CNS. Specifically, these medications block either the function of an inflammatory mediator or the entrance of immune cells into the CNS. However, these medications are facing at least 2 major challenges. First, the inflammatory mediators and immune cells, which are being blocked, are also necessary for immune defense. Consequently, current therapies compromise immunity, leading to severe side effects (*e.g.,* infections and cancers) (3, 4). Second, the therapeutic effect *via* blocking of molecules and cells is usually transient. Accordingly, frequent administration of these medications is necessary, which further compromises immunity.

To tackle these challenges, one of the vigorously pursued therapies is a myelin-specific therapy that aims to adoptively transfer or actively induce myelin-specific regulatory T (T_reg_) cells (5–9). The rationale is that the myelin-specific T_reg_ cells can specifically block the immune-mediated damage of the myelin sheath and thereby do not compromise global immune defense mechanisms (10), and potentially differentiate into memory T_reg_ cells and thereby provide a long-lasting therapeutic effect (11, 12). In this regard, one such myelin-specific therapy is a tolerogenic dendritic cell (TolDC) which, when pulsed with a myelin antigen, can induce myelin-specific T_reg_ cells (13–15). It has been shown that myelin-specific T_reg_ cells are deficient in patients with MS (16–18). Therefore, TolDC is a promising myelin-specific therapy for MS. However, recent data suggest that an *ex vivo*–generated TolDC is unstable in a proinflammatory environment and can be converted into a disease-worsening immunogenic DC (19–21).

We propose a novel engineered DC that augments myelin-specific immune regulation and works with immunogenic DCs (hence, without the *in vivo* instability concern). Specifically, this engineered DC carries an overexpressed enzyme [*i.e.,* 25-hydroxyvitamin D 1α-hydroxylase (hereafter 1α-hydroxylase)] that, under physiologic conditions, synthesizes the active vitamin D metabolite 1,25-dihydroxyvitamin D [1,25(OH)_2_D] (22). Because it is well known that an activated DC homes to the peripheral lymphoid tissues (23–26), we reason that the 1α-hydroxylase-overexpressing cytochrome P450 family 27 subfamily B member 1 (CYP27B1)-transduced DC (DC-CPY), upon *in vivo* administration, would home to the peripheral lymphoid tissues where it *de novo* synthesizes 1,25(OH)_2_D. We further speculate that this continuous *de novo* synthesis will allow the DC-CYP, within its lifespan, to create and maintain a focally high 1,25(OH)_2_D concentration at the DC-T-cell interface (or immune synapse) in the peripheral lymphoid tissues (27). Consequently, the following outcome ensues: *1*) the T cell differentiates into a stable T_reg_ cell because 1,25(OH)_2_D has been shown to induce regulatory property in T cells, (28–30) and to up-regulate the expression of Helios, which is necessary for maintaining stable regulatory properties in T_reg_ cells (28, 29, 31); and *2*) the DC is converted into a tolerogenic DC, and such tolerogenic status will be maintained within its *in vivo* lifespan, because both the *de novo* synthesized 1,25(OH)_2_D and the newly primed T_reg_ cell may tolerize the DC-CYP (32–35). Accordingly, our hypothesis is that a myelin-antigen-pulsed DC, when engineered to overexpress the 1α-hydroxylase and administered *in vivo*, homes to the peripheral lymphoid tissue where it *de novo* synthesizes the required high 1,25(OH)_2_D concentration at the DC-T-cell interface to program stable myelin-specific immune regulation. This study tested this hypothesis.

## MATERIALS AND METHODS

### Animals

C57BL/6 mice (B6, female, 6–8 wk of age, 18–20 g) were obtained from The Jackson Laboratory (Bar Harbor, ME, USA) and housed in a specific pathogen-free animal facility at Loma Linda University (LLU). Animals were allowed an acclimation of a minimum of 5 d before any experimentation. All experiments were performed in compliance with an Institutional Animal Care and Use Protocol approved by LLU Animal Care and Use Committee.

### Cell lines

DC2.4 is a bone-marrow–derived DC line kindly provided by Dr. Kenneth L. Rock (University of Massachusetts Medical Center, Worcester, MA, USA) (36).

### Fluorescence-activated cell sorting

Expressions of cell surface and intracellular proteins were analyzed by fluorescence-activated cell sorting (FACS). In brief, ∼0.5–1 × 10^6^ cells in 100 μl FACS buffer (PBS containing 1% fetal bovine serum and 0.05% sodium azide) were stained with fluorescence-conjugated antibodies specific for the desired cell surface proteins at 4°C for 30 min. The surface-stained cells were fixed and permeabilized with commercial fixation and permeabilization buffers. The cells were then stained with fluorescence-conjugated antibodies specific for the desired intracellular proteins at 4°C for 30 min in the permeabilization buffer (*e.g.,* Perm/Wash buffer; BD Biosciences, San Jose, CA, USA). Finally, the cells were washed twice in the permeabilization buffer and twice in the FACS buffer before being analyzed on a FACSAria II cytometer (BD Biosciences). Antibodies used in this study included: APC-CD3 (clone 17A2), PerCP-CD4 (clone RM4-5), FITC-IL-4 (clone BVD6-2442), FITC-IL-5 (clone TRFK5), FITC-IL-13 (clone Ebio13A), FITC-IL-10 (clone JESS-16E3), PE-foxp3 (clone FJK-16S), all from Thermo Fisher Scientific (Waltham, MA, USA), and PE-CCR7 (clone 4B12; BioLegend, San Diego, CA, USA).

### Lentivirus production and titration

Three lentiviral transfer plasmids were used in this study. One was the lentiviral mCYP-GFP gene vector (lenti-mCYP-GFP) that carried a murine CYP27B1 gene for encoding mouse 1α-hydroxylase and a GFP gene for encoding green fluorescence protein (GFP). Another was the lentiviral GFP gene vector (lenti-GFP). The third was the lentiviral h-CYP gene vector (lenti-hCYP) that carried a human CYP27B1 gene for encoding human 1α-hydroxylase. To generate a lentivirus that carried one of the lentiviral transfer plasmids, 293T cells were cultured in complete DMEM [10% fetal bovine serum (FBS), 100 U/ml penicillin/streptomycin, 3.125 × 10^−5^ M 2-mercaptoethanol, 1 mM sodium pyruvate, 0.1 mM nonessential amino acid, and 2 mM l-glutamine]. When the cells reached 70–80% confluence, the culture medium was replenished, and a transfection solution containing an envelope, a packaging, and a transfer plasmid was added drop-wise to the cells. The cells were then cultured at 37°C and 5% CO_2_ for 24 h. Then, the transfection solution was replaced with fresh DMEM containing 4% FBS, 100 U/ml penicillin/streptomycin, and 20 mM HEPES. After the cells had been cultured at 37°C and 5% CO_2_ for 48 h, supernatants were collected, filtered through a 0.45 μm filter, and centrifuged at 4800 *g* at 4°C for 24 h. The virus pellet was reconstituted in PBS containing 5% glycerol and titrated using a GFP-based FACS method. The typical titer of a virus was 10^8^–10^9^ transducing units (TU)/ml.

### Generation of primary bone-marrow–derived DCs and transduction of DCs with a lentivirus

Bone marrow mononuclear cells were isolated from mouse tibia and femur. The cells (1 × 10^6^ cells/ml) were cultured in a complete RPMI1640 culture medium containing 100 U/ml recombinant murine granulocyte–macrophage colony-stimulating factor (GM-CSF) and 10 U/ml murine Il-4 (Peprotech, Rocky Hill, NJ, USA) in a 6-well plate at 37°C and 5% CO_2_. Forty-eight hours later, nonadherent cells were gently removed and the remaining adherent clusters were further cultured. After 48 h, nonadherent cells, which were highly enriched for DCs (>90% CD11c^+^), were harvested for lentivirus transduction. In brief, 1 × 10^6^ DCs/well were cultured in a total volume of 0.5 ml culture medium containing 50 μl virus (multiplicity of infection = 40) and 8 μg/ml protamine in a 6-well plate. Twenty-four hours later, the virus was removed and the culture medium replenished. The cells were cultured for another 24 h and examined for transduction efficiency under a fluorescence microscope. If necessary, the above transduction procedure was repeated one more time. Twenty-four hours after the final virus removal, the DCs were activated by LPS (100 ng/ml for primary DCs and 1 μg/ml for DC2.4 cells) before being used for experiments.

### Real-time PCR

Forty-eight hours after the final lentivirus transduction, DCs were collected, and total RNA was isolated using the RNeasy Micro Kit (Qiagen, Valencia, CA, USA) according to the manufacturer’s instruction. First-strand cDNA was synthesized with the SuperScript III Reverse Transcriptase (Applied Biosystems, Foster City, CA). Real-time quantitative PCR was performed and analyzed in an ABI 7900HT Real-Time PCR system. Human CYP27B1 gene was amplified using the Fast SYBR Green Master Mix (Applied Biosystems) and 1 μM primers (forward: 5′-ACCCGACACGGAGACCTTC-3′; reverse: 5′-ATGGTCAACAGCGTGGACAC-3′). In addition, glyceraldehyde 3-phosphate dehydrogenase (GAPDH) was used as the housekeeping gene (forward: 5′-AATCCCATCACCATCTTCCA-3′; reverse: 5′-TGGACTCCACGACGTACTCA-3′). The PCR condition was 10 min at 95°C followed by 40 cycles of 10 s at 95°C and 15 s at 60°C. The relative expression level of human CYP27B1 gene was determined by the ΔΔ*C_t_* method and normalized to GAPDH (27).

### Determination of the 1α-hydroxylase enzymatic activity

Twenty-four hours after the final lentivirus transduction, 25-hydroxyvitamin D [25(OH)D] at the concentration of 2.5 μM was added to the culture medium. The cells were then cultured for another 24 h, and supernatants were collected for the quantification of 1,25(OH)_2_D level by a radioimmunoassay (RIA; Heartland Assays, Ames, IA, USA).

### Lymph node and spleen immunohistochemistry

After being flushed with cold PBS, spleens and lymph nodes were fixed in 4% paraformaldehyde. The fixed tissues were then embedded in an optimal cutting temperature (OCT) compound and mounted on a cryostat for sectioning. The tissues were cut into 10-µm-thick frozen sections, which were collected by adhering to slides and stored at −20°C. The tissue sections were stained with a FITC-conjugated goat anti-GFP polyclonal antibody (ab6662; Abcam, Cambridge, MA, USA) and counterstained with DAPI (Sigma-Aldrich, St. Louis, MO, USA). Images were taken with an LSM510 confocal microscope (Zeiss, Jena, Germany).

### Spinal cord histologic analysis

Spinal cord cryosections of 10 µm thickness were stained with Luxol Fast Blue (Abcam). In brief, the cryosections were stained in Luxol Fast Blue overnight at 56°C and washed with 95% ethanol followed by distilled water to remove excess stain. The color was subsequently differentiated in a lithium carbonate solution for 30 s followed by rinsing in 70% alcohol for 30 s and in distilled water for another 30 s. The sections were then examined to determine whether the white matter was clearly distinguishable from the gray matter under a microscope. When the differentiation process was completed, the sections were counterstained in a cresyl violet solution for 30–40 s, rinsed in distilled water, and differentiated in 95% ethyl alcohol for 5 min (the sections were again examined under a microscope for clear differentiation). Finally, the sections were passed twice in 100% alcohol (5 min for each pass) and twice in fresh xylene (5 min for each pass) and mounted in Cytoseal 60 (Thermo Fisher Scientific).

### Experimental allergic encephalomyelitis induction

For the induction of experimental allergic encephalomyelitis (EAE), C57BL/6 mice were immunized subcutaneously with 200 μl emulsion that contained 240 μg myelin oligodendrocyte glycoprotein peptide 35–55 (MOG_35–55_) in complete Freund adjuvant (CFA). The emulsion was made by mixing a PBS solution containing 2.4 mg/ml MOG_35–55_ with the CFA at a 1:1 ratio. The CFA was made by adding heat-inactivated *Mycobacterium tuberculosis* H37Ra (Difco Laboratories, Detroit, MI, USA) into an incomplete Freund adjuvant at the concentration of 2.5 mg/ml. At d 0 and 2, each mouse was administered 150 ng pertussis toxin (EMD Millipore, Darmstadt, Germany) intraperitoneally. The animals were clinically scored and weighed on a daily basis. The following scores were used to evaluate the severity of paralytic disease: 0, no paralysis; 1, limp tail; 2, limp tail and weak gait; 3, hindlimb paralysis; 4, forelimb paralysis and moribund (the animals will be euthanized beyond stage 4); and 5, death.

### Determination of cytokine levels in a culture supernatant

Cytokine levels in a culture supernatant were determined using the BD Cytometric Beads Array kit (BD Biosciences, San Jose, CA, USA) according to the manufacturer’s instructions.

### Statistical analysis

Statistical analysis was performed using Prism software (GraphPad, La Jolla, CA, USA). A 2-tailed Student’s *t* test was used to analyze normally distributed data. Results were considered significant when *P* < 0.05. All error bars represent the standard deviation.

## RESULTS

### Bone-marrow–derived DCs can be engineered to overexpress the 1α-hydroxylase to *de novo* synthesize a high 1,25(OH)_2_D concentration

**Figure 1.**
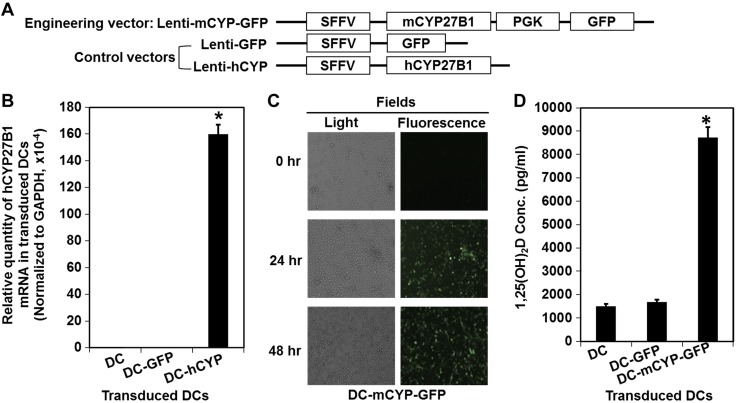
DCs can be transduced to efficiently overexpress 1α-hydroxylase to *de novo* synthesize a significantly elevated 1,25(OH)_2_D concentration. Bone marrow cells were cultured in a medium containing GM-CSF and IL-4 for differentiation into DCs. The DCs were then transduced with the indicated lentiviral vectors at d 4 and 6 during *in vitro* cultures. *A*) Lentiviral vectors used in this study included lenti-mCYP-GFP that carried a murine CYP27B1 (mCYP27B1) gene and a GFP gene; lenti-GFP that carried a GFP gene; and lenti-hCYP that carried a human CYP27B1 gene (hCYP27B1). SSFV and PGK were promoters. *B*) DCs were transduced with no vector (DC), lenti-GFP (DC-GFP), or lenti-hCYP (DC-hCYP). At 48 h after the final transduction, the DCs were then collected and analyzed for the expression of hCYP27B1 mRNAs by real-time PCR. The hCYP27B1 gene was used for distinguishing transgenic and endogenous CYP27B1 genes. Data are presented as means ± sd. *P < 0.05; Student’s *t* test (*n* = 5). *C*) At 24 and 48 h after the transduction of DCs with the lenti-mCYP-GFP (DC-mCYP-GFP), GFP protein that displayed green fluorescence was examined under a fluorescence microscope. *D*) DCs were transduced with no vector (DC), lenti-GFP (DC-GFP), or lenti-mCYP-GFP (DC-mCYP-GFP). At 24 h after the final transduction, 1α-hydroxylase substrate [*i.e.,* 24(OH)D] was added. The cells were then cultured for another 24 h, and the supernatants were collected for the quantification of 1,25(OH)_2_D level by RIA. Data are presented as means ± sd. *P < 0.05, Student’s *t* test (*n* = 6).

To test our hypothesis, we generated 3 lentiviral vectors (**Fig. 1*A***). These were lenti-mCYP-GFP, lenti-GFP, and lenti-hCYP, as described in Materials and Methods. First, we asked whether CYP27B1 mRNA was overexpressed in the lenti-hCYP-transduced DCs (DC-hCYP cells). In this experiment, we used human CYP27B1 gene to distinguish exogenous transgenic from endogenous CYP27B1 gene. To address this question, DCs were generated from bone marrow and transduced with either the lenti-GFP or the lenti-hCYP. Forty-eight hours later, the transduced DCs were analyzed for the expression of human CYP27B1 mRNA by real-time PCR. Our data demonstrated that the DC-hCYP but not the lenti-GFP-transduced DCs (DC-GFP cells) and the parental DCs expressed a significantly elevated level of human CYP27B1 mRNA (Fig. 1*B*). Second, we asked whether the 1α-hydroxylase protein was overexpressed in the lenti-mCYP-GFP-transduced DCs (DC-mCYP-GFP cells). To address this question, DCs were transduced with the lenti-mCYP-GFP and examined under a fluorescence microscope (Fig. 1*C*). Our data showed that the transduced DCs universally displayed green fluorescence 24 and 48 h after the virus transduction, indicating the overexpression of GFP and the 1α-hydroxylase proteins. Third, we asked whether the overexpressed 1α-hydroxylase was enzymatically functional. To address this question, DCs were transduced with either the lenti-GFP or the lenti-mCYP-GFP. Twenty-four hours after the final transduction, the 1α-hydroxylase substrate 25(OH)D was added to the cell cultures. The cells were then cultured for another 24 h, and supernatants were collected for the quantification of the 1α-hydroxylase enzymatic product 1,25(OH)_2_D (Fig. 1*D*). Our data demonstrated that the DC-mCYP-GFP cells produced a significantly elevated level of 1,25(OH)_2_D, as compared to the parental DCs and the DC-GFPs. In summary, we have demonstrated that primary DCs can be successfully engineered to *de novo* synthesize a high 1,25(OH)_2_D concentration.

**Figure 2.**
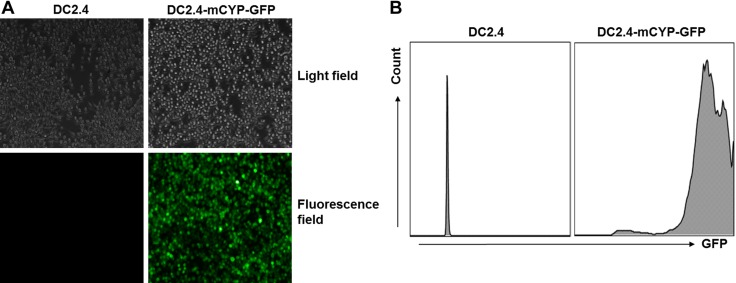
Generation of a stable bone-marrow–derived DC line that constitutively overexpresses 1α-hydroxylase. DC2.4, a bone-marrow DC line, was transduced with lenti-mCYP-GFP to generate a stable DC line, DC2.4-mCYP-GFP, that constitutively overexpressed 1α-hydroxylase and GFP. *A*) Data showed comparison of the parental DC2.4 and the DC2.4-mCYP-GFP cells under light and fluorescence fields. *B*) The expression of GFP was compared between the parental DC2.4 and the DC2.4-mCYP-GFP cells.

In addition, to facilitate addressing the hypothesis, we also took advantage of the DC2.4 bone marrow DC line, which had been shown to mimic the function of primary DCs with high fidelity (25, 36, 37). We hence asked whether a DC2.4 cell line with stable overexpression of 1α-hydroxylase could be generated. To address this question, DC2.4 cells were transduced with lenti-mCYP-GFP for multiple rounds. Our data showed that all the lenti-mCYP-GFP-transduced DC2.4 (DC2.4-mCYP-GFP) cells displayed green fluorescence under a fluorescence microscope (**Fig. 2*A***). This high-level GFP expression was further confirmed by FACS analysis (Fig. 2*B*). The DC2.4-mCYP-GFP cells could be stably expanded in cell cultures, and it was a convenient tool for us to analyze the role of DC-derived high 1,25(OH)_2_D concentration in the augmentation of myelin-specific immune regulation in the peripheral lymphoid tissues.

Furthermore, to evaluate whether the 1α-hydroxylase-overexpressing DC-CYP cells have an *in vivo* instability concern, all the DCs used in this study were not subject to tolerogenic induction and were activated by LPS.

### DC-CYP cells retain lymphoid-tissue–homing capacity

**Figure 3.**
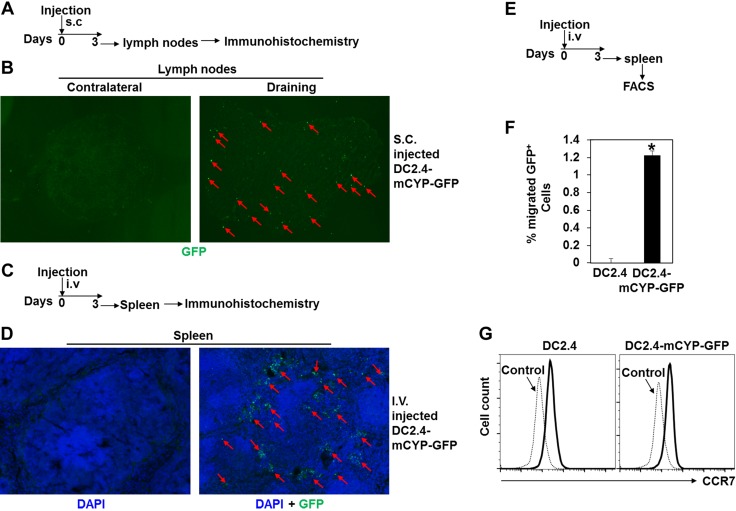
DC-CYP cells retain lymphoid tissue-homing capacity. *A*) Animals subcutaneously received 1 × 10^6^ DC2.4-mCYP-GFP cells. Three days later, draining and contralateral lymph nodes from the animals were analyzed for the presence of GFP^+^ cells by immunohistochemistry. *B*) Representative images of the contralateral and draining lymph nodes that were stained for GFP. Arrows: GFP^+^ cells. *C*) Animals intravenously received 1 × 10^6^ DC2.4-mCYP-GFP cells. Three days later, spleens from the animals were analyzed for the presence of GFP^+^ cells by immunohistochemistry. *D*) Representative images of the spleens that were stained for GFP (green). Arrows: GFP^+^ cells. *E*) Animals intravenously received either 2 × 10^6^ DC2.4 or 2 × 10^6^ DC2.4-mCYP-GFP cells. Three days later, GFP^+^ cells in the spleens were enumerated by FACS. *F*) Cumulative data showed percentage of GFP^+^ cells that migrated into the spleens. Data are presented as means ± sd. *P < 0.05; Student’s *t* test (*n* = 5). *G*) DC2.4 and DC2.4-mCYP-GFP cells were analyzed for the expression of CCR7 by FACS. Representative FACS plots are shown.

It has been well documented that both primary DCs and DC2.4 cells, when administered *in vivo*, migrate into the peripheral lymphoid tissues (23–25). We hence asked whether overexpression of 1α-hydroxylase in DCs affected this migration. To address this question, we subcutaneously administered DC2.4-mCYP-GFP cells. Three days later, contralateral and draining lymph nodes were examined for the expression of GFP by immunohistochemistry (**Fig. 3*A***). The data showed that a substantial number of the DC2.4-mCYP-GFP cells migrated into the draining but not the contralateral lymph nodes (Fig. 3*B*). In addition, we intravenously administered DC2.4-mCYP-GFP cells (Fig. 3*C*). Our data demonstrated that a significant amount of the DC2.4-mCYP-GFP cells successfully migrated into the spleen at d 3 after the injection (Fig. 3*D*). Furthermore, we asked what fraction of the DC2.4-mCYP-GFP cells migrated into the peripheral lymphoid tissues. To address this question, mice intravenously received either DC2.4 or DC2.4-mCYP-GFP cells. Three days later, GFP^+^ cells were enumerated by FACS (Fig. 3*E*). Our data showed that about 1.2% of the DC2.4-mCYP-GFP cells migrated into the spleens at the time of examination (Fig. 3*F*). Finally, we asked whether the DC2.4-mCYP-GFP cells expressed the lymphoid tissue-homing receptor C-C chemokine receptor 7 (CCR7) (23, 38, 39). Indeed, we found that DC2.4 and DC2.4-mCYP-GFP cells expressed an equivalent level of CCR7 (Fig. 3*G*). In summary, we have demonstrated that DC-CYP cells retain the ability to migrate into the peripheral lymphoid tissues.

### DC-CYP cells stimulate the expression of IL-4, -5, -10, and -13 in the peripheral lymphoid tissues

**Figure 4.**
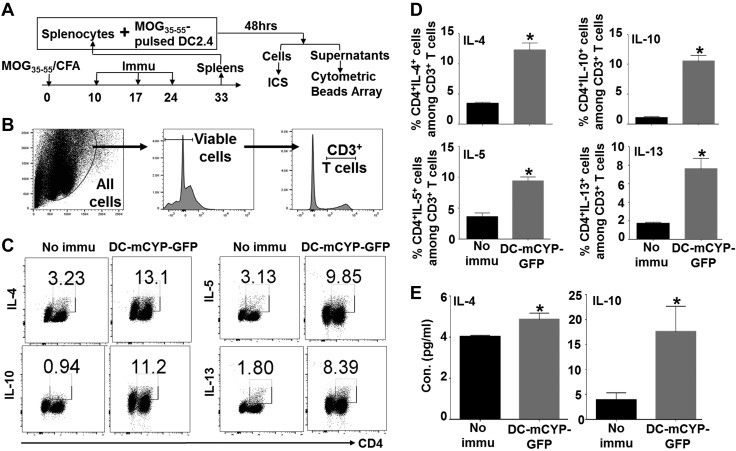
DC-CYP cells stimulate the expression of T_h_2 and Tr1 cytokines in CD4^+^ T cells. *A*) C57BL/6 mice were subcutaneously immunized with MOG_35–55_ emulsified in CFA. At d 10, 17, and 24, the animals subcutaneously received no immunization or 1 × 10^6^ DC-mCYP-GFP cells. Nine days after the final immunization, splenocytes from the animals were stimulated with MOG_35–55_-pulsed DC2.4 cells. Forty-eight hours later, the cells were analyzed by intracellular cytokine staining (ICS), and the supernatants were analyzed by cytometric bead array. *B*) Gating strategy. *C*) Representative FACS plots show the expression of IL-4, IL-10, IL-5, and IL-13 in CD4^+^ T cells. *D*) Cumulative data show CD4^+^ IL-4^+^, -10^+^, -5^+^, and -13^+^ cells among CD3^+^ T cells. Bar graphs are presented as means ± sd. *P < 0.05; Student’s *t* test (*n* = 5). *E*) Cumulative data of IL-4 and -10 concentrations in the culture supernatants. Data are presented as means ± sd. Immu, immunized; no immu, no immunization. *P < 0.05; Student’s *t* test (*n* = 5).

Upon arrival at the peripheral lymphoid tissues, a DC routinely interacts with and attempts to program and reprogram T cells. Depending on the signals that the DC provides, the T cells differentiate into functionally different subsets: T_h_2 cells that secrete IL-4, -5, and -13 (40, 41), as well as regulatory T type 1 (Tr1) cells that secrete IL-10 (42). Because both T_h_2 and Tr1 cells have been proposed to be beneficial to patients with MS (43–45), we asked whether the DC-mCYP-GFP cells programmed the secretion of these cytokines in the peripheral lymphoid tissues. To address this question, C57BL/6 mice were immunized with MOG_35–55_ emulsified in CFA to activate MOG_35–55_-specific CD4^+^ T cells. At d 10, 17, and 24, the animals subcutaneously received either no immunization or DC-mCYP-GFP cells. Nine days after the final immunization, splenocytes of the animals were stimulated with MOG_35–55_-pulsed DC2.4. Forty-eight hours later, the cells were analyzed by intracellular cytokine staining and the culture supernatants by a cytometric bead array assay (**Fig. 4*A***). Our data showed that CD4^+^ T cells from the DC-mCYP-GFP-immunized animals, as compared to the controls, secreted significantly increasing amounts of IL-4, -5, -13, and -10 (Fig. 4*B*–*D*). In addition, the culture supernatants of splenocytes from the animals immunized with DC-mCYP-GFP cells, as compared to the controls, contained significantly increasing amounts of IL-4 and -10 (Fig. 4*E*).

### DC-CYP cells program foxp3^+^ T_reg_ cells in the peripheral lymphoid tissues

**Figure 5.**
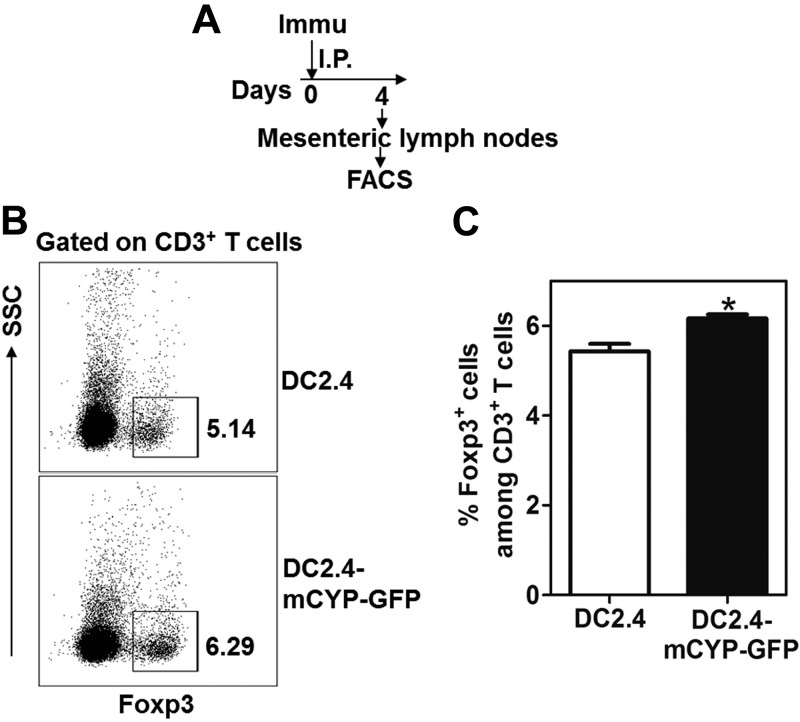
DC-CYP cells stimulate foxp3^+^ T_reg_ cells in the peripheral lymphoid tissues. *A*) BALB/c mice intraperitoneally received one of the following immunizations (immu): parental DC2.4 or DC2.4-mCYP-GFP cells. Four days later, mesenteric lymph nodes were analyzed by FACS. *B*) Representative FACS plots show foxp3^+^ cells among CD3^+^ T cells. *C*) Cumulative data from *B* show percentage of foxp3^+^ cells among CD3^+^ T cells. Data are presented as means ± sd. *P < 0.05; Student’s *t* test (*n* = 6).

In addition to T_h_2 and Tr1 cells, another important cell subset that has been proposed to have therapeutic potential for MS is foxp3^+^ T_reg_ cells. Hence, we asked whether DC-CYP indeed programmed Forkhead box protein P3 (foxp3)^+^ T_reg_ cells. To address this question, BALB/c mice intraperitoneally received the following immunizations (Fig. 5*A*): DC2.4 or DC2.4-mCYP-GFP cells. Because DC2.4 cells are in C57BL/6 background, immunization of BALB/c mice with DC2.4 stimulates a strong H-2^d^ (BALB/c) anti-H-2^b^ (C57BL/6) allogeneic response, which facilitates the detection of newly programmed foxp3^+^ T_reg_ cells. Four days later, mesenteric lymph nodes were analyzed for the expression of foxp3 among CD3^+^ T cells. Our data showed that there were significantly more foxp3^+^ cells among CD3^+^ T cells in the DC2.4-mCYP-GFP–immunized as compared to the DC2.4-immunized animals (Fig. 5*B*, *C*).

### Immunization with MOG_35–55_-pulsed DC-CYP cells provides MOG_35–55_-specific suppression of ongoing experimental allergic encephalomyelitis

**Figure 6.**
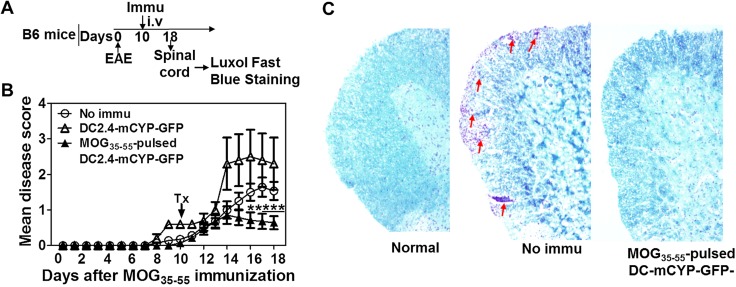
Immunization with MOG_35–55_-pulsed DC-CYP cells but not parental DCs suppresses ongoing EAE. *A*) C57BL/6 mice were immunized with MOG_35–55_ for the induction of EAE. Ten days later, the animals intravenously received one of the following immunizations: no immunization, 1 × 10^6^ DC2.4-mCYP-GFP cells, or 1 × 10^6^ MOG_35–55_-pulsed (100 μM for 3 h) DC2.4-mCYP-GFP cells. The animals were then monitored for paralytic symptoms daily. At d 18, spinal cords from the animals were analyzed by Luxol Fast Blue staining. *B*) Data show mean disease score over the observation period and are presented as means ± sd. The experiment was repeated twice with similar results. Representative data are shown. **P < 0.01, ***P < 0.001, no immu *vs.* MOG_35–55_-pulsed DC2.4-mCYP-GFP; Student’s *t* test (*n* = 5). *C*) Representative images of the spinal cord tissue sections that were stained with Luxol Fast Blue. Arrows: inflammation foci. Immu, immunized; no immu, no immunization.

Our data have now demonstrated that DC-CYP cells program T_h_2, Tr1, and foxp3^+^ T_reg_ cells. Accordingly, we reason that such DC-CYP can be potentially used for the augmentation of myelin-specific immune regulation for the treatment of EAE and MS. To address this potential, C57BL/6 mice were immunized with MOG_35–55_ emulsified in CFA for the induction of EAE. At d 10, the animals received one of the following immunizations (**Fig. 6*A***): no immunization, DC2.4-mCYP-GFP cells, or MOG_35–55_-pulsed DC2.4-mCYP-GFP cells. At d 18, the spinal cords were stained with Luxol Fast Blue for the analysis of demyelination and inflammation. Our data showed that immunization with the MOG_35–55_-pulsed DC2.4-mCYP-GFP but not the parental DC2.4-mCYP-GFP cells significantly ameliorated the paralytic disease (Fig. 6*B*). In addition, we further showed that animals without immunization showed multiple foci of inflammation and demyelination, whereas animals immunized with the MOG_35–55_-pulsed DC2.4-mCYP-GFP cells displayed fewer inflammation foci and demyelination (Fig. 6*C*).

### DC-CYP-cell-mediated suppression of EAE depends on foxp3^+^ T_reg_ cells

**Figure 7.**
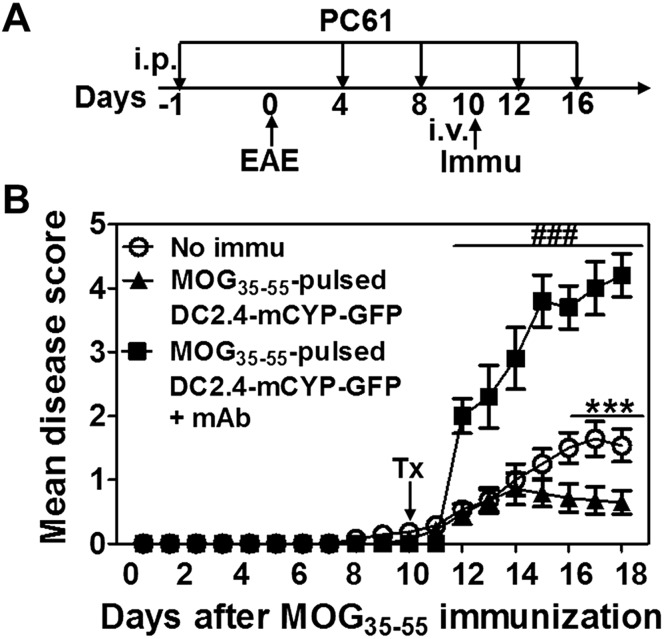
DC-CYP-cell-mediated suppression of EAE depends on foxp3^+^ T_reg_ cells. *A*) C57BL/6 mice were immunized with MOG_35–55_ for the induction of EAE. At d 10, the animals intravenously received no immunization or 1 × 10^6^ MOG_35–55_-pulsed DC2.4-mCYP-GFP cells. In addition, half of the animals that received the MOG_35–55_-pulsed DC2.4-mCYP-GFP cells also received intraperitoneal injections of an anti-CD25 mAb (clone PC61, 500 μg/mouse/injection) at d −1, 4, 8, 12, and 16 (MOG_35–55_-pulsed DC2.4-mCYP-GFP+mAb). The animals were then monitored for paralytic symptoms daily. *B*) The data show mean disease score over the observation period. Data are presented as means ± sd. ***P < 0.001, no immu *vs.* MOG_35–55_-pulsed DC2.4-mCYP-GFP; ^###^*P* < 0.01, MOG_35–55_-pulsed DC2.4-mCYP-GFP+mAb *vs.* No immu and MOG_35–55_-pulsed DC2.4-mCYP-GFP; Student’s *t* test (*n* = 5). The experiment was repeated twice with similar results. Representative data are shown. Immu, immunized; no immu, no immunization.

Next, we proceeded to ask which cell subset was essential for EAE suppression after immunization with the MOG_35–55_-pulsed DC-CYP cells. To address this question, we decided first to deplete foxp3^+^ T_reg_ cells using an anti-CD25 mAb (clone PC61), which had been shown to specifically deplete foxp3^+^ T_reg_ cells (46, 47). Accordingly, C57BL/6 mice were immunized with MOG_35–55_ emulsified in CFA for the induction of EAE (**Fig. 7*A***). In addition, the animals received one of the following immunizations at d 10: no immunization or MOG_35–55_-pulsed DC2.4-mCYP-GFP cells. Among the animals that received the MOG_35–55_-pulsed DC2.4-mCYP-GFP cells, half of the animals also received intraperitoneal injection of the anti-CD25 mAb at 500 μg/mouse per injection at d −1, 4, 8, 12, and 16 for the depletion of foxp3^+^ T_reg_ cells (MOG_35–55_-pulsed DC2.4-mCYP-GFP+mAb). Our data showed that immunization with MOG_35–55_-pulsed DC2.4-mCYP-GFP cells significantly ameliorated the paralytic disease (Fig. 7*B*). Furthermore, administration of the anti-CD25 mAb not only abrogated the therapeutic effect of the MOG_35–55_-pulsed DC2.4-mCYP-GFP cells, but also worsened the paralytic disease. Therefore, our data have demonstrated that programming of foxp3^+^ T_reg_ cells is necessary for myelin-antigen-pulsed DC-CYP cells to provide myelin-specific suppression of EAE.

## DISCUSSION

In this study, we have shown that DC-CYP cells augment the induction of T_h_2, Tr1, and foxp3^+^ T_reg_ cells in the peripheral lymphoid tissues. In addition, we have demonstrated that immunization with myelin-antigen-pulsed DC-CYP cells leads to myelin-specific suppression of ongoing EAE, which depends on foxp3^+^ T_reg_ cells. Because this novel strategy works for immunogenic DCs, it does not have the instability concern that is associated with TolDC-mediated myelin-specific therapy for multiple sclerosis (MS). Therefore, our data suggest that DC-CYP cells are a promising myelin-specific therapy for MS.

With respect to MS therapy, one of the major therapeutic goals is specifically to halt the immune-mediated damage to the myelin sheath, while sparing the global mechanism of immune defense. In principle, myelin-specific therapy is a logical strategy to achieve this goal. However, several obstacles need to be addressed before a myelin-specific therapy can be reliably implemented in clinical practice. A significant barrier associated with myelin-specific therapy is the instability of *ex vivo*-generated therapeutic cellular agents in an *in vivo* proinflammatory environment in patients with MS. An example of such cellular agents is the *ex vivo*-induced myelin-specific T_reg_ cells (induced T_reg_ or iT_reg_ cells). It has been shown that such myelin-specific iT_reg_ cells can be adoptively transferred into a host for enhancing myelin-specific immune regulation (15, 28–32). Another example is the *ex vivo*-generated TolDCs. It has been demonstrated that myelin-antigen–pulsed TolDCs can induce myelin-specific T_reg_ cells *in vivo* (19). Both cellular agents are promising therapies because patients with MS are deficient in T_reg_ cells (16–18). However, both the iT_reg_ cells (48–53) and the TolDCs are not stable after being administered to patients with MS (15,29–33). In this regard, this study has begun to address the instability concern associated with TolDCs. Indeed, we have demonstrated that even immunogenic DC-CYP cells, when pulsed with MOG_35–55_, effectively suppressed ongoing EAE (Fig. 6*A*, *B*). Because this strategy does not depend on tolerogenic status of DCs, it circumvents the *in vivo* instability concern.

We also investigated the mechanisms by which the myelin-antigen-pulsed DC-CYP cells suppressed EAE. Our data demonstrate that this disease suppression depends on foxp3^+^ T_reg_ cells (Fig. 7). These findings are consistent with recent reports that 1,25(OH)_2_D at a sufficiently high concentration can promote foxp3 expression through the binding of vitamin D receptor (VDR) to the promoter region of foxp3 (30). However, it is not yet known whether the foxp3^+^ T_reg_ cells, which are augmented by the DC-CYP cells, have memory property. Generation of memory T_reg_ cells is critical for a complete cure because neuroinflammation is perpetuated in patients with MS. In this regard, the potential generation of memory T_reg_ cells by DC-CYP cells is indeed supported by recent reports showing that 1,25(OH)_2_D stimulates the expression of a T_reg_-stabilizing molecule (*i.e.,* Helios) (28, 29, 31).

Theoretically, the foxp3^+^ T_reg_ cells, which are augmented by the myelin-antigen–pulsed DC-CYP cells, may function inside and outside the CNS. However, recent data suggest that such T_reg_ cells may predominantly function outside the CNS. These data showed that 1,25(OH)_2_D impaired CNS homing of CD4^+^ T cells by suppressing the expression of a CNS-homing receptor, C-X-C chemokine receptor 3 (CXCR3) (54). In this regard, we also observed an ∼10% reduction in the expression of CXCR3 in CD4^+^ T cells in the animals that were immunized with DC-CYP cells. However, the difference was not statistically significant (*P* = 0.67; Supplemental Fig. S1). In addition, another study showed that 1,25(OH)_2_D induced a skin-homing receptor (*i.e.,* C-C chemokine receptor 10 or CCR10) in T cells (55). Therefore, T cells stimulated by DC-CYP cells appear to be redirected away from the CNS, which makes our novel DC strategy an even more attractive therapy for MS. Further studies are needed to address this potential advantage.

In addition to the induction of foxp3^+^ T_reg_ cells, other biologic properties of 1,25(OH)_2_D also support DC-CYP cells as an attractive therapeutic agent for patients with MS. First, 1,25(OH)_2_D does not indiscriminately suppress immunity (56) and therefore is superior to other agents that also induce T_reg_ cells but broadly suppress immunity (*e.g.,* TGF-β and IL-10). Second, 1,25(OH)_2_D stimulates an antimicrobial response and hence can potentially prevent infections in patients with MS (57). Therefore, our DC-CYP strategy could lead to a paradigm shift in the myelin-specific therapy for MS.

**Figure 8.**
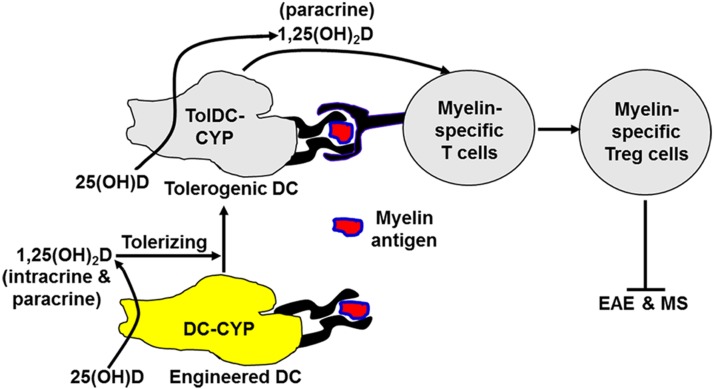
A model of myelin-specific EAE suppression mediated by DC-CYP cells. In this model, a myelin-antigen–pulsed DC-CYP cell, upon *in vivo* administration, specifically migrates into the peripheral lymphoid tissues, where it interacts with myelin-specific T cells and at the same time actively *de novo* synthesizes a high 1,25(OH)_2_D concentration at the interface of the DC-CYP and the myelin-specific T cells. Subsequently, the DC-CYP cell is self-tolerized and becomes a TolDC-CYP cell through the intracrine and paracrine action of 1,25(OH)_2_D. In addition, the myelin-specific T cells differentiate into T_reg_ cells through the paracrine action of 1,25(OH)_2_D. Consequently, the newly induced myelin-specific T_reg_ cells provide myelin-specific suppression of EAE. We further speculate that DC-CYP cells are also a promising myelin-specific therapy for MS.

Accordingly, we propose a novel model for myelin-specific suppression of EAE (**Fig. 8**). In this model, a myelin-antigen-pulsed DC-CYP cell, upon *in vivo* administration, specifically migrates into the peripheral lymphoid tissues, where it interacts with myelin-specific T cells and at the same time actively *de novo* synthesizes a high 1,25(OH)_2_D concentration at the interface of the DC-CYP and the myelin-specific T cells. Subsequently, the DC-CYP cell is self-tolerized and becomes a TolDC-CYP cell through the intracrine or paracrine action of 1,25(OH)_2_D. In addition, the myelin-specific T cells differentiate into T_reg_ cells through the paracrine action of 1,25(OH)_2_D. Consequently, the newly induced myelin-specific T_reg_ cells provide myelin-specific suppression of EAE. We speculate that DC-CYP cells are also a promising myelin-specific therapy for MS.

## Abbreviations

1α-hydroxylase: 25-hydroxyvitamin D 1α-hydroxylase
1,25(OH)_2_D: 1,25-dihydroxyvitamin D
25(OH)D: 25-hydroxyvitamin D
CFA: complete Freund’s adjuvant
CYP27B1: cytochrome P450 family 27 subfamily B member 1
CYP: CYP27B1
DC: dendritic cell
DC-CYP: CYP27B1-transduced DC
DC-GFP: lenti-GFP-transduced DC
DC-hCYP: lenti-hCYP-transduced DC
DC-mCYP-GFP: lenti-mCYP-GFP-transduced DC
DC2.4-mCYP-GFP: lenti-mCYP-GFP-transduced DC2.4
EAE: experimental allergic encephalomyelitis
FBS: fetal bovine serum
Foxp3: forkhead box protein P3
GFP: green fluorescent protein
GM-CSF: granulocyte–macrophage colony-stimulating factor
iT_reg_: induced regulatory T
lenti-GFP: lentiviral GFP gene vector
lenti-hCYP: lentiviral human CYP27B1 gene vector
lenti-mCYP-GFP: lentiviral murine CYP27B1–GFP gene vector
MOG_35–55_: myelin oligodendrocyte glycoprotein peptide 35–55
MS: multiple sclerosis
RIA: radioimmunoassay
TolDC: tolerogenic dendritic cell
TolDC-CYP: tolerogenic DC-CYP
T_h_: T helper
T_reg_: regulatory T
Tr1: regulatory T type 1
VDR: vitamin D receptor
